# Addressing a Critical Voice in Clinical Practice: Experiences of Nursing Students, Teachers, and Supervisors—A Qualitative Study

**DOI:** 10.3390/nursrep14020061

**Published:** 2024-03-29

**Authors:** Ingrid Rachel Strand, Unni Knutstad, Anton Havnes, Mette Sagbakken

**Affiliations:** Faculty of Health, OsloMet—Oslo Metropolitan University, 0130 Oslo, Norway; unnikn@oslomet.no (U.K.); anton@oslomet.no (A.H.); metsa@oslomet.no (M.S.)

**Keywords:** clinical practice, student–supervisor relationship, asymmetry in power, critical reflection

## Abstract

Aim: Our goal was to explore how power asymmetry manifests within the relationships between students, teachers, and supervisors, and how it influences students’ ability for critical reflection. Design: This study has an explorative qualitative design. Methods: Thirty in-depth interviews with nursing students (15), teachers (9), and supervisors (6) were conducted in addition to 16 observations of mid-term assessments during clinical practice. The analysis was conducted using Braun and Clarke’s thematic analysis. Results: The students described being a student as a balancing act between humility, conforming to the supervisor’s expectations, and speaking their minds. The view expressed by the teachers and supervisors is that training for the nursing profession is closely linked to the students’ ability to act independently. Due to the supervisors’ hierarchical position, however, students are hesitant to voice any criticism regarding insufficient supervision or unsatisfactory performance of clinical tasks while at the same time being evaluated on their ability to critically reflect on their own and others’ clinical performance. This study was prospectively registered with the Norwegian Centre for Research Data on the 15th of August 2017 with the registration number 54821.

## 1. Introduction

Nursing students in Norway undergo a period of supervised clinical practice in their bachelor programme. They are supervised by a Registered Nurse (RN) in clinical practice and a faculty member from the university (teachers). The teachers are either assistant professors or associate professors in the bachelor program in nursing at the university. The supervisors are RNs; employees either in clinical wards, hospitals, nursing homes, home-based care, or psychiatric units; and not employees at the university. The supervisors play a crucial role in evaluating the students` clinical competence and readiness for independent practice. Approval from a supervisor or teacher (or both) is usually necessary for students to progress in their clinical training or to pass their clinical rotations. This evaluation process is important to ensure that the students have the knowledge, skills, and critical thinking abilities required to provide safe and high-quality patient care. Clinical competence is defined by Liou et al. [1] as “The ability to apply critical thinking, problem solving and clinical decision-making skills in patient care” [1] p. 2654). We lean on the definition of Griffiths and Tann on critical reflection, where they argue that critical reflection can be both spontaneous and a more systematic reflection [2]. The significance of being able to assess and reflect on one’s clinical competencies, as well as those of others, is well documented in research as a critical factor in enhancing patient safety [3,4,5]. Thus, critical thinking is seen as a crucial part of clinical competence in nursing. However, a challenge highlighted in prior research is that the capacity for self-reflection and assessment typically grows with experiences, a quality that nursing students pursuing a bachelor’s programme may possess to varying degrees [6]. Previous studies focusing on students’ learning experiences in clinical practice have identified several factors that influence their learning process. These include the necessity of a close and trustful relationship with their supervisors [7,8,9,10,11,12,13,14]. A safe relationship supports students’ professional development, protects, and assists them in times of difficulty, and guides their learning throughout clinical practice [8]. Honkavuo’s qualitative study of the perspective of Finnish nursing students regarding a caring relationship with their supervisor revealed that a relationship built on mutual respect and having supervisors well versed in research-based pedagogical techniques was of utmost significance. This implied that supervisors possessed insight into optimizing students’ learning experiences [8]. 

In the evaluating process, there are three defined roles: the student, the Registered Nurse in clinical practice, and the teacher. Both Rees et al. and Johnson [5,15] underline the importance of power asymmetry as manifested by defined roles. 

Power imbalances may emerge in both the student–supervisor and student–teacher relationship. In their role, both supervisors and teachers will be wielding greater authority compared to the students [5,15]. Besides defined roles, several influencing factors, including age, knowledge, experience, language proficiency, and hierarchical position, can create or increase this discrepancy in power [15]. Thus, the discrepancy can be understood as an imbalance within the relationship, often referred to as power asymmetry. 

In *Discipline and Punish: The birth of the prison*, Foucault examines the development of disciplinary mechanisms and the exercise of power within various institutions, including schools and hospitals. Foucault argues that these institutions are not merely places of education and healing, but also sites where power is exercised and normalized. He suggests that disciplinary power operates through a variety of techniques, such as surveillance, normalization, and examination, to regulate individual behaviour and enforce conformity to social norms. In the context of educational institutions, Foucault explores how disciplinary practices, including hierarchical structures, strict schedules, and surveillance mechanisms, are employed to mould students into obedient subjects [16].

In our point of view, understanding power asymmetry in this context of education and supervision is essential for ensuring fairness, accountability, and effective mentorship. This asymmetry can also influence students and supervisors’ communications and collaboration. Haugan highlights the need to address power dynamics for creating a supportive and productive learning environment, which can help the students to express their critical perspectives in various educational settings [13]. 

A study conducted in Norway concerning the student–supervisor relationship in clinical practice found that a lack of continuity and inadequate supervision led to students feeling uncertain about their own capabilities [13]. One of the primary objectives in examining the student–supervisor relationship within the context of power asymmetry is to heighten awareness of potential power-related challenges that might hinder students’ learning, such as instances where supervisors fail to provide constructive feedback. Rees emphasizes the critical need for a balanced relationship to facilitate student development and learning, while recognizing that a power imbalance could potentially obstruct effective communication and feedback [5], p. 1150. At its essence, Philling and Roth claim that supervision aims to support learners in their professional development, and supervisors can achieve this by adopting a relationship-based approach to education and training [17]. Conversely, power imbalances can lead to abuse, as seen in a study on undergraduate nursing students’ clinical experiences conducted in South Africa [10]. Examples included supervisors making disparaging comments on students’ assessment, stifling, or suppressing students’ expression and opinions, and subjecting them to verbal mistreatment in front of peers. While cultural norms may vary, promoting belongingness is crucial across contexts, empowering students to advocate for themselves, engage in self-assessment, and enhance their learning experiences.

However, both students and supervisors can wield power, and students may respond to and utilize this power in various ways. Rees et al. revealed that students might exercise their own power by, for instance, actively seeking feedback from supervisors and engaging in discussions about such feedback with both the supervisor and fellow students, particularly when they are dissatisfied with the supervisor’s communication style [5]. 

Perry et al. conducted a comprehensive review aimed at identifying factors that enhance student accountability for learning in clinical practice, including countries such as Sweden, Canada, the UK, and the USA. In their review, they established that a crucial aspect contributing to this accountability is the sense of belongingness. Belongingness, as defined by Perry et al., refers to the presence of a nurturing relationship with a supportive supervisor who fosters an environment with support throughout the learning process. Importantly, if the relationship is defined by belonging, it is also a relationship experienced as a partnership, emphasized by both Perry et al. and Dysthe as implying an exchange of reflections and ideas [18,19,20].

Several studies have explored the dynamics of student–supervisor interactions within clinical practice, spanning various countries such as Norway [7,12,13], Finland [8], Denmark [9], Australia [5], South Africa [10], England [11], and Sweden [14]. However, few studies have delved into the student–supervisor relationship’s power asymmetry and its repercussions for their interactions, including its impact on students’ ability to reflect on both their own and others’ clinical performances.

As stated above, while there is a wealth of research on the experiences of students and supervisors in clinical practice, only a limited number of studies have explored how power imbalances among students, supervisors, and teachers affect interaction patterns. This study aims to explore how power asymmetry may manifest within the relationships between students, teachers, and supervisors (RNs), and how it may influence students’ ability for critical reflection, with data from actual assessment situations and mid-term assessment.

### Research Questions

1. What are the experiences and perceptions of students, teachers, and supervisors regarding their relationships and the dynamics of power asymmetry?

2. How may potential power asymmetry influence students’ ability to critically reflect?

## 2. Method

### 2.1. Study Design

This study adopts a qualitative, exploratory design with the primary goal of enhancing our comprehension of power imbalances between students and supervisors and how it influences students’ ability to critically reflect. This research incorporates 30 in-depth interviews conducted with students, teachers, and supervisors within a bachelor’s degree program at a Norwegian university. Additionally, 16 mid-term assessment observations were included as a method to gain deeper understanding of the contextual dynamics and interactions among the involved parties [21,22].

To ensure maximum variation, inspired by Patton, this study encompassed all three academic years of the program, spanning placements in diverse healthcare settings such as homecare, nursing homes, surgery wards, medical wards, and psychiatric units. Variation was also secured by encompassing individuals of different genders and ages, as well as students, teachers, and supervisors (see Table 1) [23].

### 2.2. Setting

This study, comprising both interviews and observations, was carried out in various clinical settings where students underwent their clinical placements. The mid-term assessment (MTA) of the students served as a pivotal context for observing the interactions between the students, their program teacher, and the clinical supervisor. The MTA typically occurs approximately four to five weeks into the clinical placement and involves the student, a teacher from the bachelor programme, and the supervisor representing the clinical setting.

To ensure an uninterrupted environment, a separate room within the ward is utilized for the MTA, which typically lasts between 45 and 60 min. During the MTA, an assessment form is utilized, encompassing a list of predefined learning outcomes, with the performance being scored as “as expected” or “below expected”. This MTA can be viewed as a tripartite discussion involving the student, the teacher, and the clinical supervisor, with the student receiving both formal and summative feedback on their performance and clinical competencies related to the specified learning outcomes.

### 2.3. Recruitment

The participants for this study were recruited with the assistance of teachers from the bachelor program in nursing who served as gatekeepers. A total of 44 teachers were approached for participation in this study, either via email or during a teacher’s meeting. Thirteen teachers agreed and consented to participate. During a pre-clinical practice meeting, this study was presented to students (totalling 161) of these 13 teachers, resulting in 74 students signing consent forms. To accommodate the timing and the mid-term assessments (MTAs) and to encompass various clinical placements for data collection, 16 students were selected to participate in the observations. Subsequently, the supervisors of these 16 students were approached and invited to join this study, and they all agreed and signed consent forms. The teachers participating in this study were either assistant professors or associate professors in the bachelor program in nursing at the current university. The supervisors participating in this study were RNs; employees in either clinical wards, hospitals, nursing homes, home-based care, or psychiatric units; and not employees at the current university. We did not address whether the supervisors and/or teachers had participated in any formal education in student clinical assessment beyond their bachelor’s degree in nursing.

All the observed participants were also invited to take part in interviews, and fifteen students, nine teachers, and six supervisors agreed to do so. Teachers and supervisors who declined to participate in the interviews cited time constraints as their reason for non-participation. One student who did not participate in the interview declined due to personal challenges encountered during clinical practice.

### 2.4. Participants

To ensure maximum variation [23], we included different genders and ages, as well as a sample comprising students, teachers, and supervisors (see Table 1). The number of potential participants that received information about this study and signed the consent form totalled 100, including teachers, students, and supervisors (Table 1). However, due to organizational limits and the timing of the mid-term assessment, a strategic sample was made to include participants in all types of clinical placements. The sample included 9 teachers, 16 students, and 13 supervisors.

### 2.5. Data Collection

To gain a more comprehensive understanding of the interactions between students, teachers, and supervisors during the assessment process, observations and interviews were chosen as methods. The MTA was selected for observation due to an assumption that this setting might reveal situations where potential power imbalances in the interactions among the participants become evident. A total of 16 MTA observations were conducted, with a specific focus on the verbal and nonverbal interaction patterns among the participants. The notes and reflections from the observations were typed on a computer by the first author during the observation process.

Approximately two to three weeks after the MTA, 30 in-depth interviews were conducted in private settings, either at the university or within clinical settings. These interviews followed a semi-structured interview guide. Students were questioned about their experiences of assessment (including MTA), their relationship with their supervisor, how they perceived their role as students in clinical practice, and their overall clinical experiences. Similarly, teachers and supervisors were asked questions about their experiences in supervising and assessing students in clinical practice, including the MTA.

### 2.6. Analysis

This study is part of a larger research project that centres on the assessment of nursing students in clinical practice, exploring the experiences of students, teachers, and supervisors. For the analysis of the data, Braun and Clarke’s six-step thematic analysis framework [24] was employed.

The initial data analysis began during the transcription of the interviews, with comments and codes being recorded as interviews were transcribed and observational notes were revised. To structure and organize the text systematically, all data pages were imported into the qualitative analysis tool NVIVO, version 12 PRO. A second comprehensive reading was conducted manually, where each line of the text was carefully reviewed. Key text passages pertaining to different facts of interaction patterns during supervision and assessment in clinical practice, including potential power imbalances among students, teachers, and supervisors, and their impact on students’ ability to engage in critical reflection, were analysed using NVIVO. An inductive approach to the data characterized the initial part of the analysis.

During the coding process, the recognition of power asymmetry in the relationship among the three parties involved prompted the formulation of a more refined research question: “How may potential power asymmetry influence students’ ability to critically reflect?”. This research question guided the subsequent abductive phase of the analysis. Excerpts providing insights into the relationship, interaction, and consequences of supervisors and teachers’ methods of supervision; power asymmetry; reactions to power asymmetry; the exercise of power asymmetry; and how power asymmetry manifested in the relationship, and students’ ability to critically reflect, were assigned new codes.

The following phase involved successive rounds of condensing codes that were relevant to the research question, resulting in the emergence of patterns related to power asymmetry and its diverse impacts on the students’ ability to critical reflect. Throughout this process, themes were progressively abstracted into overarching themes. These overarching themes served as a guide to ensure consistency in all phases of the analysis and theme abstraction, aligning with the research question. Ultimately, the data were condensed into three overarching themes: (i) different expectations, (ii) socialized into servility, (iii) daring to critically reflect. The analytical process was characterized by a dynamic interplay between meaningful entities, condensed meaning units, abstraction, code development, and theme generation. During the process of analysis, codes and themes were discussed with the researchers of this study and members within the research group as a validation of findings. As this study progressed, all four authors collectively agreed upon these three final themes.

### 2.7. Ethical Considerations

This study received approval from the Norwegian Centre for Research Data in August 2017, case number 54821. This study also gained approval from the university’s department head and the program coordinators for the first, second, and third years of the bachelor program in nursing. The directors and department managers of various healthcare institutions and departments also provided their approval for this study.

All participants, including students, teachers, and supervisors, were provided with both verbal and written information about this study’s objectives and purpose. They were informed of their right to withdraw from this study at any time without facing any adverse consequences. All participants signed a consent form.

To ensure data security and privacy, a risk and vulnerability analysis was conducted. Subsequently, data were stored on an encrypted memory stick, which was placed in a locked drawer, and access was restricted to the researcher alone.

## 3. Findings 

### 3.1. Different Expectations

The results reveal that the assessment process was influenced by the individual expectations of teachers and supervisors regarding what they anticipated from the students. This encompassed their expectations of student behaviour and performance in interactions with colleagues and patients across different departments. The findings suggest that when supervisors and teachers assert influence over student conduct, these personal expectations may be perceived as a form of control or even disciplinary measures. An illustrative incident during a mid-term assessment further exemplifies this phenomenon. This pertains to an adult nursing student with relevant knowledge, who answered a professional question posed by a co-worker, a nurse. This was interpreted as arrogant behaviour and inappropriate coming from a student. The issue was brought up during the mid-term evaluation, on observation, and the following was expressed by the teacher:

*“You should be yourself, but don’t demand too much attention” (at the ward).*

Different expectations from the educational institution and from the clinical placement were illustrated by a second-year teacher during an interview:

*“In this regard, I wish that we were much more ideologically visionary and ideologically based in our education, in facing a field of practice that would be willing to play along. However, how do you get thousands of practical contacts and fields of practice to engage with us on matters they have no interest in?”*

The teacher seemed to have a specific view on the supervisors as not visionary. The supervisors, however, had some views on limits in the nursing education, as stated by a supervisor in clinical practice:

*“Perhaps the university should swallow that bitter pill and simply write it out plainly on paper, thus sparing the students from trembling like leaves and feeling as though they are about to discuss matters they do not comprehend in the slightest”.*

Although the supervisor and teacher may not intentionally communicate their expectations to express their authority, students often interpreted the communication of expectations as asymmetrical. Even though the expectations outlined by teachers and supervisors were deemed to be pertinent, the way they were conveyed was crucial. This was exemplified when a teacher, during an interview, contemplated the anticipated skills for the nursing profession while also considering the hierarchical position of the student within the ward:

*“My impression is that students, well, they may feel like they’re a nuisance, and they may feel that they cannot ask, but at the same time, I feel that it is a skill in a busy working day to be able to know when I can ask questions and when I have to wait, because not all questions require immediate answers”.*

This contemplation positions the student within a hierarchical framework, whether consciously or subconsciously, possibly serving as a means of their initiation into the profession or establishing expectations regarding student conduct. Data based on the interviews with students indicated that students often passively accepted their position as a student and their role within the ward hierarchy, conforming to supervisors’ expectations when observed. Students also expressed a sort of acceptance of the ongoing adaptation of expectations and set of standards they experienced from different supervisors in different clinical placements during their bachelor program. New clinical placements required new insights into the new supervisors’ expectations. One student commented on different expectations during their interview, and said:

*“What do you expect today? I need to know what is expected of me. Everybody has different expectations”.*

This quote reflects the frustration expressed by multiple students during clinical practice, feeling the need to conform to their supervisors’ varying expectations.

### 3.2. Socialised into Servility

Several students articulated their experience as a delicate balance between demonstrating humility, conforming to their supervisors’ expectations, and having the courage to voice their opinions. A first-year student reflected during their interview on how they were met the first day in clinical practice, and how that first meeting made them feel unimportant and inferior:

*“They were unaware that we were coming... it was a case of “oh, are you coming today... and why were we not informed?”, which resulted in an unpleasant atmosphere.”*

Furthermore, the same student described a lack of training in nursing skills during clinical practice in nursing homes, and that it was difficult to question the task and activities they were left in charge of:

*“I had expected to be given more responsibility than we were…that we would be tested more in relation to typical nursing tasks. However, we were more or less assigned a role of activity coordinators, with a great deal of focus being placed on that.”*

Similar reflections were communicated by several other students regarding their first day in clinical practice. As a result, students often found themselves feeling relegated to the bottom of the hierarchy, with their expectations and desires regarding their role often going unfulfilled. This perceived asymmetry appeared to hinder their acquisition of clinical skills, leading to a diminished sense of autonomy. In an interview, a third-year student shared the sentiment of feeling burdensome in relation to their supervisor and colleagues:

*“You often feel you are a burden […], and then there may be some people who show that it’s exhausting having students (laughs) […] I’ve just accepted that this is a part of the student’s role”.*

Most students stated that they were “aware” of their position or status in the clinical ward hierarchy, and, consequently, in their learning environment, and that this position required some degree of subservience. On the other hand, students emphasized factors such as trust and consistent feedback from their supervisor as essential elements in their relation and the development of the necessary clinical competencies. Additionally, teachers discussed the process of students assimilating into the nursing profession and how both they and their supervisors passed on knowledge and clinical skills to the students through imitation. A second-year teacher expressed this during an interview:

*“I experience way too much imitation of what’s going on at the ward […] learning is about much more than just imitation, so I would prefer to see a more active way of acquiring clinical competencies.”*

A second-year teacher connected the act of imitation to a lack of confidence in students and their approach to acquiring competencies during an interview:

*“I think it’s about a lack of trust in the student, in them being capable of finding their own way. That we sort of do a quality assurance, almost like a meat inspection, instead of thinking the opposite, giving them (the students) optimal opportunities.”*

Moreover, the data indicated that supervisors’ expectations regarding students’ adaptation to the nursing profession were linked to their capacity for independence within the clinical setting, as elucidated by a third-year supervisor during an interview:

*“What we are working towards is that they should be as independent as possible […] if they take responsibility for the patient, and several patients, and use their supervisors as a consultant, that’s great.”*

Findings derived from both observations and interviews show that both teachers and supervisors stressed the importance of students demonstrating independence in their execution of clinical tasks and activities. Nevertheless, the data also suggest that supervisors and teachers resorted to disciplinary measures to maintain control over students, while students emulated their supervisors to achieve what was perceived as vital clinical competence by the supervisor. This disparity, along with the resulting imbalance in the student–supervisor, student–teacher dynamic, appeared to be linked to supervisory and teaching roles rather than actual knowledge.

Data from the student interviews indicated that students often passively accepted their position as a student and their role within the ward hierarchy, conforming to supervisors’ expectations when observed.

During an interview, a second-year teacher conveyed this sentiment while responding to inquiries about how students were adopted into the nursing profession, supporting the perceptions of many students:

*“They (the students) are in a way socialized into a form of servility.”*

When asked about students’ integration into the nursing profession, this teacher’s response aligns with the perceptions of numerous students. The implication is that, instead of openly engaging in critical reflection, students tend to unquestioningly embrace the views and practices of their supervisors.

### 3.3. Daring to Critically Reflect 

Based on both observational and interview data, it became evident that some students hesitated to openly discuss negative perceptions or experiences related to the competence of their clinical colleagues and supervisors. They feared that expressing such viewpoints might result in not passing clinical practice. Similarly, the students were cautious about sharing critical feedback regarding the adequacy of the supervision. In an interview, a third-year student expressed fear, during an interview, of not being taken seriously if they complained about the supervision:

*“It’s one person’s words against the other—who are they going to listen to?”.*

Consequently, students refrained from expressing critiques of the leadership, supervisors’ performance in clinical tasks, and inadequate supervision due to being apprehensive about potential consequences. As articulated by a third-year student in an interview, this fear of repercussions was a significant factor in their reluctance to voice such concerns:

*“You’re afraid of speaking out as a student. I just grit my teeth and get through it” (clinical practice).*

Students described how such interactions consumed valuable time that could otherwise be dedicated to learning activities during clinical practice. They expressed concerns about not being heard or taken seriously by their teachers, supervisors, or the head of the department. Although supervisors encouraged students to improve their clinical and professional skills through self-reflection and constructive feedback, students perceived the use of such feedback as “risky”.

## 4. Discussion

In this study, we examined the experiences and perceptions of students, teachers, and supervisors regarding their relationships and the dynamics of power asymmetry in an assessment and evaluation setting. Additionally, we explored how such asymmetry might influence students’ ability to engage in critical reflection. Students frequently described challenges in expressing critical perspectives within clinical practice due to hierarchical structures, fearing potential negative reactions from supervisors. Teachers experienced that students often imitate their supervisors excessively. They attributed this behaviour to the imbalance in power dynamics in the relationship, resulting in students behaving in accordance with what they perceived as supervisors’ expectations and demands. Supervisors observed a deficiency in students’ independence in pursuing their learning outcomes.

However, they did not express specific concerns about potential consequences stemming from an imbalanced power dynamic in the student–supervisor relationship, such as students seldom critically reflecting and openly assessing and discussing performances and practices at the clinical ward.

Previous research has shown that essential nursing skills include the capacity to engage in reflective practices, the ability to critically analyse, and the evaluation of various aspects of nursing [10,25]. Other studies have identified challenges in sharing reflections, often attributed to power imbalances between teachers and students [26], or students being inhibited from expressing their opinions due to an abuse of authority [10]. Due to the hierarchical position of the supervisor, students hesitated to offer constructive feedback regarding inadequate supervision or a substandard execution of clinical duties. This discovery presents a paradox, as the desired learning outcomes emphasize the importance of students’ ability to critically reflect on their clinical experiences. According to our findings, it was the personal expectations of the individual supervisors rather than the typical ward-level expectations that primarily influenced the comments and expectations regarding students’ clinical performance. This subjective approach by supervisors regarding key clinical competencies and their emphasis on student evaluation based on these abilities makes it challenging to convey clear expectations to students before their clinical placements.

In our study, teachers noted how supervisors tended to pass on their own methods for performing clinical tasks, which was interpreted as a lack of trust in the students’ ability to develop their own learning paths. This lack of student independence aligns with Foucault’s argument that disciplinary measures often result from a lack of trust in students’ capacity to acquire new skills independently [16].

The role of a supervisor or teacher as a guide for students entering the nursing profession can also be viewed as an embodiment of tradition. In this context, traditions encompass the preservation and transmission of assessment knowledge, clinical competencies, and institutional understanding [27], p. 70. However, as our findings indicate, students’ imitation and adjustment to the established norms and practices in institutional settings could be seen as a socialization into the profession, following a traditional form of learning of the nursing profession. This again could limit their learning process rather than facilitate it, restraining the students from critical thinking and independence. When knowledge is merely handed down or passively transmitted to students, they might be prevented from actively and critically acquiring the knowledge necessary to become professional nurses. This process of imitation can inadvertently perpetuate outdated practices or even lead to malpractice. To aid students in cultivating a critical perspective on their own and their colleagues’ clinical task performance, process evaluation becomes essential, enabling students to assess their current standing and map out their course for further learning [28,29,30,31,32].

Adopting an assessment framework for the clinical learning environment, as suggested by Ozga et al., as a routine procedure for evaluating students’ experiences during clinical practice could offer valuable insights. This approach allows supervisors and educators to scrutinize and contemplate the dynamics of their supervisory relationships and the role of the nursing teacher and direct attention towards the pedagogical environment [33].

As illustrated in our findings, asymmetric relations and hierarchical systems tended to prevent students to voice concerns about what they perceived as irregularities in supervision. Consequently, our results suggest that students’ hesitancy regarding offering critical feedback on the clinical performance of supervisors or colleagues, including their supervisory abilities, may be related to teachers and supervisors’ disciplinary inclinations. These findings align with those of a Norwegian study by Christiansen et al., which revealed that supervisors’ anticipations of student conduct influenced the assessment process, including the anticipation of independence in the learning process. Additionally, it was observed that supervisors predominantly based their assessments on their personal judgments of what comprised critical nursing skills [7].

According to our study, students appear to conform and adapt to their supervisors’ current expectations, despite having encountered different standards from previous supervisors or educators. This adaptability may be attributed to students quickly adapting to their position or role within the existing hierarchy, or, as Foucault (p. 123) emphasizes, to the status or role of being a student. In institutional settings, such as hospitals or universities, shared norms and hierarchies, like the ward hierarchy, exemplify the power structures that students must acclimatize to [16]. These power dynamics can vary across different institutions [34]. In general, a doctor holds the highest status within a hospital due to superior expertise and skills [16,35]. Similarly, a nurse holds a higher rank than an auxiliary nurse, and depending on their progress in training, nursing students fall somewhere between the status of a nurse and an auxiliary nurse.

Students embark on a transformative journey to become nurses and enter the field of nursing. In this process, teachers and supervisors play a pivotal role in guiding students and providing feedback on various aspects such as their conduct, interactions with colleagues and patients in the ward, and their overall professional engagement. When supervisors, whether consciously or unconsciously, instil obedience in students, it may result in future nurses conforming to the system rather than critically evaluating and reflecting on professional standards.

Drawing on Foucault’s insight [16], the exertion of power by teachers and supervisors can subjectify students, forming them to behave in accordance with the supervisors’ expectations, essentially leading to socialization into subservience. A recognized Danish researcher who focuses on the student–supervisor relationship and feedback argues that students should rely on themselves and their own learning process to acquire the essential competencies, but self-reliance is contingent on the presence of a trusting relationship between the student and the supervisor [9]. In a parallel manner, data based on the student interviews emphasize the importance of fostering a relationship founded on mutual trust and providing trustworthy, consistent feedback. This aligns with findings from prior research, e.g., Ref. [18], highlighting the importance of providing students with opportunities for autonomy and accountability in clinical practice. It also emphasizes the necessity for supervisors to utilize pedagogical approaches that foster the cultivation of independent learning. Dysthe’s partnership model [19] offers a valuable perspective on the supervisor–student relationship. According to this model, feedback is conveyed through dialogue, allowing for an open discussion and negotiation of the feedback. Dysthe emphasizes that the relationship does not assume symmetry in terms of students and supervisors possessing identical knowledge or insight. Instead, the supervision process fosters a dialogic space where both parties can initiate and advocate for their perspectives. A partnership-oriented connection, as outlined by this model, has the potential to facilitate the development of students` critical thinking and independence, rather than attempting to assimilate the students into a traditional mould or encouraging them to imitate the supervisor. This approach can be effectively employed to rectify or balance existing asymmetry and improve the overall learning environment, as highlighted by previous research [5,9,15].

From our perspective, the use of disciplinary measures by supervisors or teachers as a form of authority may exacerbate the existing asymmetry, potentially hindering students from freely expressing themselves. However, a supervisor or teacher may find it necessary to exercise a degree of restraint and discipline to guide a nursing student’s journey into the profession, somewhat akin to providing institutional “parenting” to the student. Thus, a complete lack of disciplinary measures, without any form of quality assurance for students transitioning into the nursing profession, may pose a risk to the integrity of both the profession and the institutions where they practice. Nevertheless, it is crucial for supervisors and educators to be mindful of the potential drawbacks of asymmetry in the student–supervisor relationship, ensuring that students can fully harness their learning potential and openly articulate their critical reflections on supervision and other clinical matters.

## 5. Conclusions

In conclusion, the insight shared by teachers and supervisors underlines the close connection between training for the nursing profession, students’ critical thinking skills, and their ability to function independently. Nevertheless, the hierarchical position of supervisors appeared to inhibit students from voicing critiques concerning inadequate supervision or subpar clinical task performances, even though they were simultaneously being assessed on their capacity to critically evaluate both their own and their colleagues’ clinical performance. This apparent contradiction arises from the evaluation criteria, which entail an expectation that students actively engage in critical reflection and assessment of their co-workers and peers. Despite the potential power imbalances that could suppress or disempower students, our findings revealed that students frequently accepted their hierarchical position and adapted to their role as a student.

To gain a more comprehensive understanding of the power asymmetry in student–supervisor relationships, we recommend that future research should concentrate on institutional dynamics and involve leaders in clinical practice. Additionally, we propose conducting student and supervisor observations in diverse clinical settings, followed by in-depth interviews focusing on their perceptions of their own authority and its potential impact on others. Such investigations can provide valuable insights into the complexities of the power dynamics within the student–supervisor and student–teacher relationship.

## 6. Limitations

The data for this study were collected from one university and one bachelor program in nursing in Norway. However, due to the similarity of findings from previous studies, we believe the findings have transfer value to other nursing and health education programs in similar contexts. We acknowledge that the data utilized in this study are from 2017 and 2018. However, it is important to note that the regulation of clinical placements and associated assessment procedures has remained unchanged since 2016. There is still a need for the further exploration of assessment in clinical practice, particularly focusing on the asymmetry in the relationship between supervisors and students. This aspect remains relevant and warrants further exploration.

## Figures and Tables

**Table 1:** Participant sample (F—female, M—male).

Sample	Observed	Participated in Interview	Year of Program 1st/2nd/3rd yr.	Age Range
Teachers	9 (7F/2M)	9 (7F/2M)	3/3/3	57–70
Students	16 13F/3M	15 (11F/4M)	4/3/8	20–58
Supervisors	13 13F	6 6F	1/2/3	25–59
Total	38	30	30	20–70

## Data Availability

Dataset available on request from the authors. The raw data supporting the conclusions of this article will be made available by the authors on request.

## Public Involvement Statement

No public involvement in any aspect of this research.

## Guidelines and Standards Statement

This manuscript was drafted against the consolidated criteria for reporting qualitative research (COREQ) for qualitative research.
